# Adaptive dose escalated radiotherapy in oropharyngeal cancers: a treatment planning feasibility study

**DOI:** 10.1186/s13014-022-01991-x

**Published:** 2022-02-05

**Authors:** Laura Grocutt, Claire Paterson, Ronan M. Valentine

**Affiliations:** 1 CRUK RadNet Glasgow, University of Glasgow, Glasgow, G61 1QH UK; 2 Beatson West of Scotland Cancer Centre, NHS Greater Glasgow and Clyde, Glasgow, UK; 3 Beatson West of Scotland Cancer Centre, Radiotherapy Physics, NHS Greater Glasgow and Clyde, Glasgow, UK

**Keywords:** Head and neck cancer, Dose escalation, Treatment planning, VMAT, RapidPlan™, Multi-criteria optimisation, Simultaneous integrated boost, Response adaptive

## Abstract

**Background:**

A significant proportion of patients with poor prognosis squamous cell cancer of the oropharynx relapse loco-regionally despite radical (chemo)radiotherapy. If a predictive biomarker for disease control can be identified during treatment then individualised and adaptive treatment strategies may be employed. The aim of this study is to assess the feasibility of adaptive and dose-escalated RT to the gross tumour volume without increasing surrounding planning target volume doses and maintaining clinically acceptable organs at risk doses.

**Materials and methods:**

Twenty representative patients with poor prognosis locally advanced OPSCC who were known to have relapsed post RT, were re-planned retrospectively using Eclipse TPS v15.5, RapidPlan™ and multi-criteria optimisation. In our centre, PTV65 is treated with 65 Gy in 30 fractions while areas at risk of containing microscopic disease (PTV54) are treated synchronously to 54 Gy in 30 fractions. The original clinical plans were re-optimised to act as controls (Group I). These plans were split into two plans of 15 fractions each, with the latter 15 fractions used to escalate the dose to the GTV to 73 Gy (Group II) and 82 Gy (Group III). Plan sums were created for the total 30 fractions to record plan evaluation parameters along with assessments of plan deliverability.

**Results:**

For all groups, the dose coverage at D98% and D50% for the PTVs were comparable. The D2% dose levels for PTV65-GTV increased. All dose levels associated with PTV54 remained largely unaffected by the dose escalation regimens. Conformity indices for PTV65 and PTVAll (PTV65 plus PTV54) reveal comparable target volume coverage across all three groups. Despite the GTV being escalated by 12.3% and 26.2% in groups II and III, the volume of GTV receiving > 84 Gy was considerably less than 1.75 cc. While OAR doses increased for the escalated groups, these increases were not clinically significant.

**Conclusion:**

This planning feasibility study exploring RapidPlan™ combined with multi-criteria optimisation has demonstrated that doses to the GTV may be escalated without increasing PTV65-GTV, PTV54 or OAR doses considerably, suggesting an interventional clinical trial using this approach would be feasible.

## Introduction

Head and neck (H&N) cancers are the 6th most common cancer worldwide [1]. 95% are squamous cell carcinomas (HNSCC), originating from the epithelial mucosal lining of the upper aerodigestive tract. Around 60% of HNSCCs present with locally advanced but non-metastatic disease and are associated with poor survival outcomes [2]. The pattern of treatment failure is loco-regional; many patients go on to die from this disease without developing distant metastases [3–5]. Radical (chemo)radiotherapy ((C)RT) involving 6–7 weeks of daily RT with concurrent platinum-based chemotherapy is widely accepted as standard of care in locally advanced HNSCC.

In oropharyngeal squamous cell cancer (OPSCC) there are several ongoing international trials in treatment de-intensification for Human Papillomavirus (HPV)-positive cancers given their very good prognosis [6]. In contrast, Ang et al.’s pivotal study showed patients with high risk features including heavy smoking history and HPV-negative disease had a 3 year overall survival of only 46.2% [7]. The analysis by Ang et al. also showed that the differences in survival between the good and poor prognosis groups were largely due to differences in loco-regional control (LRC), indicating treatment intensification to improve LRC may improve outcomes significantly.

Increasing the dose of RT is an appropriate avenue to explore; there is an established dose–response relationship in HNSCC, therefore escalating the radiation dose may improve tumour control and treatment outcomes [8]. Higher rates of LRC have been demonstrated in early stage clinical trials of uniform dose-escalation with acceptable toxicity profiles [9–11]. This is yet to translate into improved disease outcomes in larger phase 3 trials e.g. the ART-DECO study in larynx and hypopharynx SCC showed no improvement in LRC with dose-escalated intensity modulated radiotherapy (IMRT) [12]. It must be noted that this uniform approach to dose-escalation has only been applied to entire poor prognostic groups as described above. An individualised dose-escalation strategy using a predictive biomarker of response during treatment could be used to select the sub-group of patients for whom intensified treatment is most beneficial. This may allow us to realise the full potential of RT dose-escalation.

Adaptive radiotherapy can be divided into two distinct categories; anatomy adapted and response adapted [13]. In this study we have focused on the latter scenario. Response adapted radiotherapy consists of changing the target volumes and/or the doses depending on the response to treatment. Functional imaging techniques such as PET-CT and fMRI are under investigation as imaging biomarkers of response in H&N cancer and may enable response adapted radiotherapy to become a feasible paradigm [14, 15].

Dose escalation was previously difficult to implement in HNSCC with conventional radiotherapy techniques because of the proximity of organs at risk (OARs) to target volumes and increased risk of radiation-induced toxicity. However, with advances in radiotherapy technologies, such as IMRT, highly conformal dose distributions and improved sparing of OARs can be achieved. Eclipse™ v15.5 has advanced features that give the user more control and speed during the planning process. RapidPlan™ (RP) and the Multi-Criteria Optimisation (MCO) features can offer the user additional solutions during the plan optimisation stage. RP is a knowledge based algorithm, which estimates dose volume histogram curves and suggests optimisation objectives with priorities for OARs for the current plan by extracting pertinent information from previously optimised plans. During MCO, a library of “Pareto optimal” plans is generated, automatically highlighting different trade-off objectives. The user can vary the clinical criteria, navigating across the Pareto surface, exploring the variations between optimal OAR sparing and tumour target volume coverage to create a final deliverable treatment plan.

The aim of this study was to investigate the feasibility of adaptive, dose-escalated radiotherapy in patients with poor prognosis OPSCC, increasing dose to gross tumour volume (GTV) while maintaining dose to surrounding planning target volumes (PTVs) and nearby OARs.

## Materials and methods

### Patient selection

Eligible patients were those with locally advanced poor prognosis oropharyngeal SCC [7] who had received radical primary RT or CRT. Patients had participated in the MeRInO study (study of diffusion weighted MRI as a predictive biomarker of response during radiotherapy for high and intermediate risk squamous cell cancer of the oropharynx) [5] and those selected for this sub-study were participants who were known to have loco-regional relapse. Research ethics committee approval was gained for the primary study (reference 15/WS/0159) and written informed consent was obtained for each patient with specific permission requested to use their imaging dataset for additional research beyond the primary study.

### Target volume and OAR delineation

GTV was delineated for primary tumour and involved lymph nodes. A 10–15 mm margin was added, then the outline further edited to exclude natural barriers to spread e.g. bone and air cavities, and extended to include the whole involved nodal level(s) to create the clinical target volume (CTV65). CTV54 included nodal areas considered at risk of containing microscopic disease as per international guidelines [16]. A 3 mm geometric expansion was used to create the planning target volumes (PTV65 and PTV54). A dose of 65 Gy in 30 fractions over 6 weeks was prescribed to PTV65 and 54 Gy to elective areas (PTV54). Pertinent OAR outlining consisted of the larynx (from hyoid to cricoid), ipsi and contra-lateral parotids, spinal cord and brainstem. Planning organ at risk volumes (PRVs) of 3 mm were added to the spinal cord and brainstem.

### Treatment planning

#### Planning objectives

Table 1 details the planning constraints set for PTV coverage and OAR sparing. While OAR constraints for this study were in line with the Phase 3 PARSPORT trial [17], PTV constraints were slightly stricter by ensuring 98% of the prescribed dose to 95% of the PTV volumes as per ICRU 83 [18].

**Table 1:** Dose-volume planning objectives for target volumes and organs at risk

Structure	Dose-volume planning objective
PTV65 (65 Gy prescribed in 30 fractions);	99% volume more than 90% of the dose (D99% > 90%) 98% volume more than 95% of the dose (D98% > 95%) 50% volume equal to 100% of the dose (D50% = 100%) 5% volume less than 105% of the dose (D5% < 105%) 2% volume less than 107% of the dose (D2% < 107%)
PTV54 (54 Gy prescribed in 30 fractions);	99% volume more than 90% of the dose (D99% > 90%) 98% volume more than 95% of the dose (D98% > 95%) 50% volume equal to 100% of the dose (D50% = 100%) 5% volume less than 117% of the dose (D5% < 117%) 2% volume less than 122% of the dose (D5% < 122%)
PRV Spinal Cord	1% of volume less than 44 Gy; (D1% < 44 Gy) Dmax less than 48 Gy
PRV Brainstem	1% of volume less than 48 Gy (D1% < 48 Gy)
Ipsi-lateral Parotid	As low as reasonably practicable
Contra-lateral Parotid	Dmean less than 24 Gy
Larynx	Dmean less than 40 Gy

GTV; dose-volume planning objectives similar to as described for PTV65 but for an overall escalated prescribed dose of 73 Gy in 30 fractions or 82 Gy in 30 fractions

PTV65 was used as a surrogate of mucosa and as such a mandatory constraint of no more than 1.75 cc of GTV was allowed to receive > 84 Gy, this having previously been shown to be the dosimetric threshold above which late grade 4 mucosal ulcers will develop [19]. Remaining OAR and target volume dosimetry was considered along with clinical context by an experienced head and neck clinical oncologist to determine acceptability. Table 2 shows the three fractionation schedules investigated. Plan 1 represents the dose per fraction for the first 15 fractions, while Plan 2 signifies the dose per fraction for the remaining 15 fractions. PTV65 and PTV54 received 2.17 Gy per fraction and 1.80 Gy per fraction, respectively in all plans and all dose cohorts, the change in dose per fraction was applied only to GTV. Group I received standard dose and fractionation, 2.17 Gy per fraction for plan 1 and 2. Group II received 2.17 Gy per fraction in Plan 1 and 2.70 Gy per fraction in Plan 2. Group III received 2.17 Gy per fraction in Plan 1 and 3.30 Gy per fraction in Plan 2.

**Table 2:** Details of the three groups, each with alternative dose and fractionation schedules, employed in this study

	Group I (n = 20)	Group II (n = 20)	Group III (n = 20)
Total Dose	65 Gy/30 fractions	73 Gy/30 Fractions	82 Gy/30 Fractions
EQD2 Gy_α/β=10_	65.92 Gy_10_	75.82 Gy_10_	87.82 Gy_10_

Target volume		Dose/fraction (Gy)	Fractions	Dose/fraction (Gy)	Fractions	Dose/fraction (Gy)	Fractions
PTV65	Plan 1	2.17	1–15	2.17	1–15	2.17	1–15
GTV		2.17	1–15	2.17	1–15	2.17	1–15

Total dose		32.5 Gy/15 fractions		32.5 Gy/15 fractions		32.5 Gy/15 fractions	
PTV65	Plan 2	2.17	16–30	2.17	16–30	2.17	16–30
GTV		2.17	16–30	2.70	16–30	3.30	16–30

The physical dose along with the biological dose is tabulated

#### Radiobiological calculation

The radiobiological calculations determined for this treatment planning feasibility study using the EQD2 equation [20] lie within a range previously published from a collection of dose escalation studies [21]. Equivalent doses in 2 Gy per fraction (EQD2) were calculated for each of the study groups using an α/β ratio = 10 for early responding tissues. EQD2 Gy values were calculated individually for the plan 1 and plan 2 plans, which were then subsequently combined for each group. For group I plans; 65 Gy in 30 fractions resulted in a total dose of 65.92 Gy_10_ (EQD2 Gy), for group II plans; 73 Gy in 30 fractions resulted in total dose of 75.82 Gy_10_ (EQD2 Gy) and finally for group III plans; 82 Gy in 30 fractions resulted in total dose of 87.82 Gy_10_ (EQD2 Gy).

#### Planning technique

The Eclipse™ treatment planning system (TPS) Varian Medical Systems, Palo Alto, Ca, v15.5 was used to create the treatment plans. An interactive dose-volume optimiser is used to define and fine-tune the desired doses to the PTVs and close lying structures and compute an optimal plan for the patients which best achieves the stated dose while minimising the dose to the OARs. Eclipse™ uses photon optimiser (PO) for plan optimisation and Acuros® XB advanced dose calculation algorithm v15.5.7 for dose calculation. All radiotherapy plans were generated using a grid size of 2.5 mm. A 6 MV photon energy and 600 MU/min dose rate was used for all Volumetric Arc Therapy (VMAT) plans utilising two full coplanar arcs with the collimator rotated to 30 degrees for clockwise rotation and 330 degrees for counter clockwise rotation. Collimator jaw tracking was applied to each plan. For each plan re-optimisation, our departmental Head & Neck RP model was used in conjunction with MCO [22].

#### Plan optimisation

Group I plans were re-optimised first; the RP model was used to generate DVH estimates which then translated into optimisation objective parameters, which were applied during the optimisation process. An experienced planner modified the parameters as necessary to achieve an optimal plan. Once an optimal plan solution was achieved, the planner then selected MCO for objective trade-off exploration. Each dose objective associated with PRV spinal cord, PRV brainstem, ipsi- and contra- lateral parotids and larynx was selected individually for trade-off exploration. Upper and lower point dose objectives were chosen for PTV65 and PTV54. The dose distribution was evaluated according to the clinical planning objectives (Table 1). Once acceptable, plan re-optimisation for group II and group III then commenced; the group I plans were subsequently split into Plan 1 and Plan 2 plans consisting of 15 fractions each. As described above, the Plan 1 plans are identical (e.g., 32.5 Gy in 15 fractions) across the three groups but additional optimisation objectives were added to the GTV for the Plan 2 plans in group II and III. For example, the group II plans had GTV escalated to 73 Gy in the Plan 2 plan while using the Plan 1 plan as a base dose plan in the optimiser. Once the GTV was escalated with an acceptable solution, MCO was selected and the additional upper and lower point dose objectives were chosen for the GTV. Trade-off exploration was launched once again in an effort to further reduce OAR doses. The plans for each group were added together into a plan sum following final dose calculation and the clinical objectives were assessed. The dose was prescribed to the median dose as recommended by the ICRU 83; in other words, all plans were normalised so that the prescription dose to PTV65 was 65 Gy ± 1 Gy. Furthermore, the GTV was escalated to 73 Gy ± 1 Gy and 82 Gy ± 1 Gy for the group II and III plans, respectively. One experienced planner was responsible for undertaking all of the treatment planning tasks while another experienced planner assumed the role of checking all plans.

### Analysis of plans

#### Dose volume histogram parameters

The plans in each group were quantitatively compared by DVH analysis. To evaluate the irradiated dose to the OARs, PTVs and the GTV, the analysis involved comparisons to the constraints presented in Table 1.

#### Plan evaluation parameters

For PTV65, PTV54 and GTV, the conformity index (CI) and homogeneity index (HI) were recorded for each group. Furthermore, we introduce a new conformity index; high dose fall-off index (HDFI). The CI is an indicator that is used to assess target volume coverage together with the extent of normal tissue sparing. In this study, we have defined the CI as outlined by Eq. 1, which is a modified version of the prescription dose spillage equation defined in the most recent SABR consortium guidelines [23]. Unlike SABR, which uses a 100% reference isodose volume, we recorded a more study appropriate 98% reference isodose volume for the PTVs (CI) and 107% reference isodose volume for the GTV (HDFI).

1 $${\text{CI}}_{{({\text{xn}})}} = {\text{Body}}\;\left( {{\text{V}}98\%_{{({\text{yn}})}} } \right)/{\text{TV}}_{{{\text{xn}}}} \left( {{\text{V}}98\%_{{({\text{yn}})}} } \right);$$

where x_1_ = PTV65 and y_1_ = 65 Gy prescription dose, x_2_ = PTVAll and y_2_ = 54 Gy prescription dose, Body = volume of patient receiving at least 98% of the prescription dose, TV = target volume receiving at least 98% of the prescription dose.

The HDFI quantitatively describes the dose fall-off from a boost region (e.g., GTV) fully encompassed by a larger PTV. HDFI’s were calculated for group II and group III plans by recording the V107% of the 65 Gy prescription dose inside and surrounding the GTV with specific ring structures/growth margins applied. V107% was chosen given its importance as an upper planning objective for H&N VMAT plans.

2 $${\text{HDFI}}_{{({\text{GTV x}})}} = {\text{Body}}\;\left( {{\text{V}}107\%_{{(65{\text{ Gy}})}} } \right)/{\text{GTVx}}\;\left( {{\text{V}}107\%_{{(65 {\text{Gy}})}} } \right);$$

where x = 0 mm, 5 mm or 10 mm margin surrounding the GTV, Body = volume of patient receiving at least 107% of the prescription dose, 65 Gy, GTV = volume of GTV receiving at least 107% of the prescription dose, 65 Gy.

For CI and HDFI; 1 is the ideal value representing better conformal coverage.

The HI is a metric ratio used to analyse the uniformity of the dose distribution in the target volume. In this study, we have defined the HI as.

3 $${\text{HI}}_{{({\text{x}})}} = {\text{D}}5\%_{{({\text{x}})}} /{\text{D}}95\%_{{({\text{x}})}} ;$$

where x = target volume of GTV, PTV65 or PTV54.

For HI; 1 is the ideal value representing better dose homogeneity.

Furthermore, the volume of the body receiving 6 Gy (V_6__Gy_), 12 Gy (V_12__Gy_), 24 Gy (V_24__Gy_) and 48 Gy (V_48__Gy_) were recorded as a measure for scatter dose. Finally, the V107%_(65__Gy)_ was recorded for an additional three structures, namely, Body–GTV, Body–(GTV + 5 mm) and Body–(GTV + 10 mm). The V107%_(65__Gy)_ recorded for the Body–(GTV + 5 mm) structure and Body–(GTV + 10 mm) structure were in turn divided by the V107%_(65__Gy)_ recorded for the Body–GTV structure. Consequently, the V107%_(65__Gy)_ within each of these structures enabled the dose fall-off (or percentage decrease) as a function of distance outside the GTV to be ascertained for each group.

#### Plan deliverability

To examine plan deliverability, quality assurance (QA) measurements were performed on a Varian TrueBeam™ linear accelerator equipped with millennium Multi Leaf collimator (MLC) (60 leaf pairs, maximum leaf speed of 2.5 cm/s, maximum gantry speed of 6 degrees/s and variable dose rate up to 600 MU/min). A comparison between the planned dose and delivered dose was performed using the Mapcheck2 phantom device setup on the treatment couch. A 2D global gamma analysis together with an acceptance of 95% points passing the criteria of 3 mm for the distance to agreement (DTA) and a dose difference tolerance level of 3% was employed. In addition to the machine QA performed, RadCalc v6.3 was used to re-calculate each plan offering an independent MU check along with other complexity parameters such as the modulation factor (MF) (defined as the ratio of MU required at a reference point with dynamic MLC to the MU required at a reference point in an open field) and average leaf pair opening (ALPO) (defined as the average leaf pair opening at each control point with a weight assignment proportional to the number of MUs).

### Statistical analysis

The Shapiro–Wilk test determined that our study data was normally distributed. Consequently, all statistical analyses between the three groups were performed using the ANOVA test followed by the Bonferroni post-hoc test. The threshold for statistical significance was, therefore, set to *p* < 0.0167; a *p*-value less than 0.02 was considered significant.

## Results

**Fig. 1:**
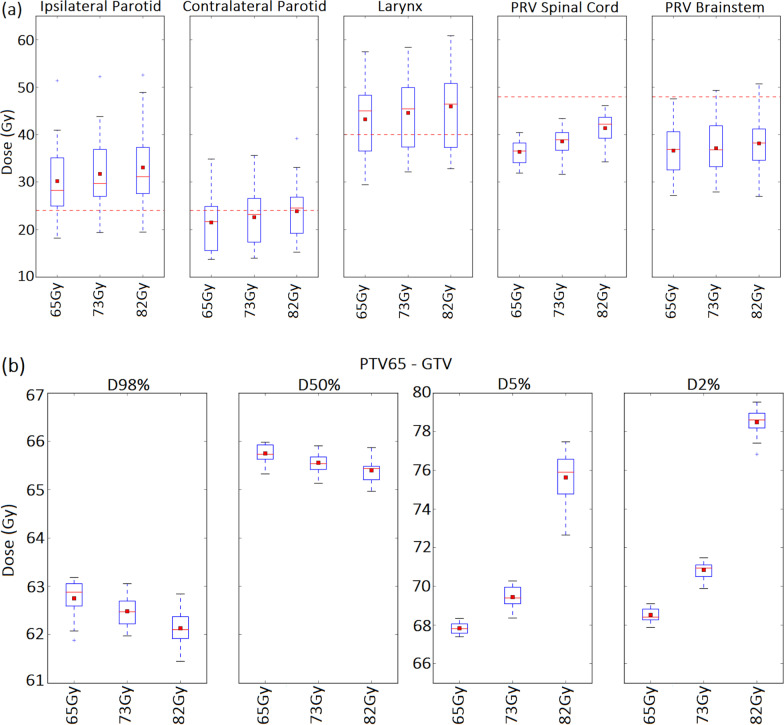
Box plot of the mean absolute doses for **a** all pertinent OARs and for **b** PTV65-GTV in each of the study groups (n = 20). The mean value is indicated by the red square symbol, the median value by the central horizontal line, the interquartile range is represented by the box, and the outliers are indicated by the asterisks. The dose constraints are highlighted in **a** by the red horizontal dotted line

20 patients were included in the study. On average the plans had a mean (± standard deviation) PTV65 volume of 367.9 cc (± 101.4 cc), PTV54 volume of 272.4 cc (± 84.8 cc) and GTV volume of 19.8 cc (± 11.6 cc). Figure 1a presents the mean absolute doses for the pertinent OARs as a function of study group. As the dose is escalated to group II and group III plans, OAR doses increase accordingly. Although there was a 12.3% and 26.2% prescription dose increase to the GTV between group I & group II, and group I & group III, respectively, the percentage dose increases to the pertinent OARs were considerably less, ranging from 1.4% to 6% in group II and 4.1% and 13.7% in group III. Figure 1b illustrates the mean absolute doses for PTV65-GTV for all study groups. All radiotherapy plans achieved the lower planning objectives (D98%) for the target volumes. Furthermore, the median dose, D50% equivalent to 65 Gy ± 1 Gy was accomplished for PTV65 among the three groups. The median dose to the GTV in the escalated groups was within ± 1 Gy of their respective prescriptions. The D2% for PTV65-GTV, PTV65 and GTV saw a notable percentage increase in the escalated groups (group II; 3.8% (PTV65-GTV), 7.4% (PTV65), 10.8% (GTV), and group III; 14.9% (PTV65-GTV), 20.3% (PTV65), 24.1% (GTV)) compared to the standard group (Fig. 1b and Table 3). Furthermore, we found the volume of GTV receiving > 84 Gy to be considerably less than 1.75 cc (group I; V84 Gy = 0.0 ± 0.0: group II; V84 Gy = 0.0 ± 0.0: and group III; V84 Gy = 0.3 ± 0.5). All PTV54 constraints, i.e., D98%, D50%, D5% and D2% were achieved for each of the three groups and found to be within a 1% variation.

**Table 3:** Lists plan evaluation parameters and %difference/statistical results for GTV, PTV65 and PTV54 across all three groups

Parameters	Group I (65 Gy)	Group II (73 Gy)	Group III (82 Gy)	I vs II	I vs III
Dose-volume values (Gy)	Mean ± SD (95% CI)	Mean ± SD (95% CI)	Mean ± SD (95% CI)	% Diff (*p*-value)	% Diff (*p*-value)
*Targets*					
GTV (D98%)	64.0 ± 0.4 (63.8–64.2)	71.9 ± 0.4 (71.7–72.0)	80.0 ± 0.8 (79.6–80.3)	+ 12.3% (*p* < 0.02)	+ 25.0% (*p* < 0.02)
GTV (D50%)	65.6 ± 0.5 (65.4–65.8)	73.8 ± 0.3 (73.7–73.9)	82.6 ± 0.2 (82.5–82.7)	+ 12.5% (*p* < 0.02)	+ 25.9% (*p* < 0.02)
GTV (D2%)	67.5 ± 0.5 (67.3–67.8)	74.8 ± 0.2 (74.7–74.9)	83.8 ± 0.4 (83.6–83.9)	+ 10.8% (*p* < 0.02)	+ 24.1% (*p* < 0.02)
PTV65 (D98%)	62.8 ± 0.4 (62.6–62.9)	62.6 ± 0.3 (62.5–62.7)	62.2 ± 1.2 (61.6–62.8)	− 0.3% (*p* = 0.19)	− 1.0% (*p* < 0.02)
PTV65 (D50%)	65.8 ± 0.2 (65.7–65.8)	65.7 ± 0.2 (65.6–65.8)	65.6 ± 0.2 (65.5–65.7)	− 0.3% (*p* = 0.28)	− 0.3% (*p* < 0.02)
PTV65 (D2%)	68.5 ± 0.4 (68.3–68.7)	73.6 ± 0.9 (73.2–74.0)	82.4 ± 1.1 (81.9–82.9)	+ 7.4% (*p* < 0.02)	+ 20.3% (*p* < 0.02)
PTV54 (D98%)	51.5 ± 0.4 (51.3–51.7)	51.8 ± 0.3 (51.7–52.0)	52.0 ± 0.5 (51.8–52.3)	+ 0.6% (*p* < 0.02)	+ 1.0% (*p* < 0.02)
PTV54 (D50%)	54.9 ± 0.5 (54.7–55.1)	55.2 ± 0.5 (55.0–55.5)	55.4 ± 0.6 (55.2–55.8)	+ 0.5% (*p* = 0.05)	+ 1.0% (*p* < 0.02)
PTV54 (D2%)	60.4 ± 0.8 (60.1–60.8)	60.4 ± 0.7 (60.0–60.7)	60.5 ± 0.7 (60.2–60.8)	0.0% (*p* = 0.81)	+ 0.1% (*p* = 0.80)

GTV, gross target volume; PTV65, planning target volume receiving 65 Gy; PTV54, planning target volume receiving 54 Gy; 95% CI, 95% confidence interval; Mean ± SD, Mean ± Standard Deviation; % Diff, Percentage Difference

### Dose metrics

Similar CI _(PTV65)_ and CI _(PTVAll)_ where found between the three groups (Table 4). Superior GTV dose homogeneity, HI _(GTV)_ was attained for group II; 1.03 ± 0.01 followed by group III; 1.04 ± 0.01 and group I; 1.05 ± 0.01. Comparing the range of dose volumes, it can be shown that the dose splash or scatter dose in the body at each selected dose level is not significantly different between the three groups (6 Gy, *p* > 0.02; 12 Gy, *p* > 0.02; 24 Gy, *p* > 0.02; 48 Gy, *p* > 0.02).

**Table 4:** Outlines the conformity and homogeneity indices for each group

Parameters	Group I (65 Gy)	Group II (73 Gy)	Group III (82 Gy)	I vs II	I vs III
Dose metric values	Mean ± SD	Mean ± SD	Mean ± SD	% Diff (*p*-value)	% Diff (*p*-value)
CI_(PTV65)_	1.07 ± 0.03	1.06 ± 0.03	1.05 ± 0.03	− 0.9% (*p* = 0.17)	− 1.9% (*p* = 0.15)
CI_(PTVAll)_	1.38 ± 0.09	1.41 ± 0.08	1.42 ± 0.08	+ 2.2% (*p* = 0.33)	+ 2.9% (*p* = 0.37)
HI _(PTV65)_	1.07 ± 0.01	1.14 ± 0.03	1.27 ± 0.05	+ 6.5% (*p* < 0.02)	+ 18.7% (*p* < 0.02)
HI _(PTV65-GTV)_	1.07 ± 0.01	1.10 ± 0.01	1.20 ± 0.02	+ 2.8% (*p* < 0.02)	+ 12.1% (*p* < 0.02)
HI _(GTV)_	1.05 ± 0.01	1.03 ± 0.01	1.04 ± 0.01	− 1.9% (*p* < 0.02)	− 1.0% (*p* < 0.02)
HI _(PTV54)_	1.12 ± 0.02	1.11 ± 0.01	1.12 ± 0.01	− 0.9% (*p* = 0.60)	0.0% (*p* = 0.62)

CI, conformity index; HI, homogeneity index

For comparison of the high dose fall-off outside the GTV boost volume, HDFI’s were calculated for the escalated plans only. We found the Body/GTV_0mm_ ratio to increase from 2.05 ± 0.54 for group II to 4.79 ± 2.00 for group III (*p* < 0.02). Considering GTV plus a 5 mm margin, the ratio between the body receiving 107% of 65 Gy and this structure also increased from 1.06 ± 0.09 for group II to 1.50 ± 0.30 for group III (*p* < 0.02). Applying a 10 mm margin around the GTV for comparison with the body, we found the V107% to be comparable and close to 1 for both groups (group II; 1.06 ± 0.09 and group III; 1.04 ± 0.06; *p* = 0.57), indicating that the dose has fallen off to equivalent levels 10 mm beyond the GTV. In fact for group II, the dose fall-off was equally close to 1, 5 mm beyond the GTV emphasising an initial sharper fall-off associated with the lower escalated dose of 73 Gy.

Further to the calculation of HDFI’s, the dose fall-off (or percentage decrease) as a function of distance outside the GTV was assessed. We found that for the Body–GTV, Body–(GTV + 5 mm) and Body–(GTV + 10 mm) structures, V107%_(65__Gy)_ was 3.6 cc, 3.6 cc and 3.5 cc, respectively (Group I); 17.0 cc, 2.3 cc and 2.1 cc (Group II) and; 56.8 cc, 22.6 cc and 3.4 cc (Group III). While similar V107%_(65__Gy)_ values were recorded for Group I, we found V107%_(65__Gy)_ decreased by 86.6% (Group II) and 60.2% (Group III) at a distance of 5 mm outside the GTV. At a distance of 10 mm outside the GTV, the V107% decreased by 87.6% (Group II) and 93.9% (Group III).

**Fig. 2:**
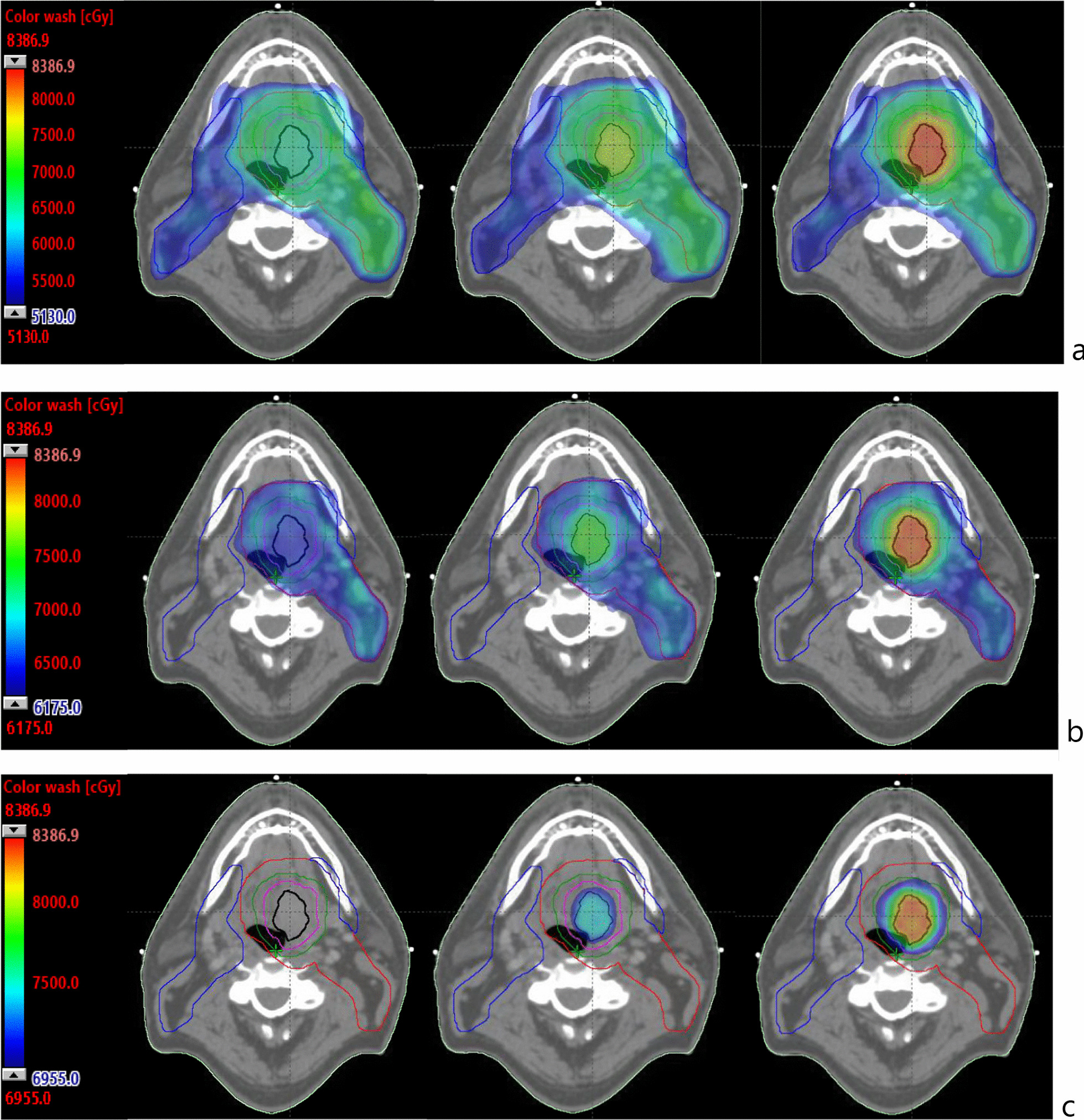
Examples of colour wash dose distributions on CT axial slices **a** 51.30 Gy (95% of 54 Gy, PTV54); **b** 61.75 Gy (95% of 65 Gy, PTV65); and **c** 69.55 Gy (107% of 65 Gy, PTV65) for a representative case in groups I (left), II (middle) and III (right). Structure colours: PTV54—blue; PTV65—red; GTV–black; GTV plus 5 mm margin—magenta; GTV plus 10 mm margin—green

The escalated dose to the GTV boost volume is clearly displayed in groups II and III emphasising the fact that the dose increase is concentrated in the vicinity of the GTV while lower dose levels remain largely unaffected beyond the GTV plus 10 mm structure. Beyond the GTV plus 10 mm margin, all plans qualitatively display similar dose coverage at the 51.30 Gy and 61.75 Gy dose levels (Fig. 2).

### Machine delivery parameters

Complexity assessments were carried out using metrics as shown in Table 5. We found that the MF and ALPO increased as the dose was escalated to the GTV, indicating a lower level of modulation. An increase in the number of MUs associated with the standard group I plans corroborates a higher plan complexity compared to the group II and III plans. Furthermore, QA measurements were performed on each plan with results again suggesting that group II and group III plans were comparable to group I plans in terms of plan calculation accuracy.

**Table 5:** Plan deliverability parameters; monitor units (MUs), average leaf pair openings (ALPO), modulation factor (MF) and global gamma analysis pass-rate for each group of plans (mean ± standard deviation)

Deliverability Metrics	Group I (65 Gy)	Group II (73 Gy)	Group III (82 Gy)	I vs II	I vs III
	Mean ± SD	Mean ± SD	Mean ± SD	% Diff (*p*-value)	% Diff (*p*-value)
MUs	752 ± 9	710 ± 7	709 ± 7	− 5.6% (*p* = 0.14)	− 5.7% (*p* = 0.12)
ALPO	2.61 ± 0.31	2.87 ± 0.24	2.90 ± 0.28	+ 10.0% (*p* < 0.02)	+ 11.1% (*p* < 0.02)
MF	0.31 ± 0.05	0.35 ± 0.05	0.38 ± 0.07	+ 12.9% (*p* < 0.02)	+ 22.6% (*p* < 0.02)
Pass Rate (%) Global	99.7% ± 0.3	99.9% ± 0.2	100% ± 0.1	+ 0.2% (*p* = 0.54)	+ 0.3% (*p* = 0.51)

MUs, Monitor Units; ALPO, Average Leaf Pair Opening; MF, Modulation Factor

## Discussion

Functional imaging during radiotherapy has the potential to identify the sub-group of patients who are not responding adequately to treatment, during treatment. The delivery of response adaptive and dose-escalated radiotherapy for those predicted to ‘fail’ treatment is crucial to the paradigm of biologically adaptive radiotherapy and this in-silico study has demonstrated the feasibility of adaptive dose-escalated radiotherapy for poor prognosis OPSCC.

Despite relatively small GTV volumes, clinically acceptable PTV and OAR doses that adhered to accepted constraints were maintained. Our main findings were that for a 12.5% (Group II) and 25.9% (Group III) increase in median dose to the GTV the associated OAR doses increased by 1.4–6.0% and 4.1–13.7%, respectively. We found no significant increase in OAR (PRV Brainstem, Ipsi-lateral and Contra-lateral Parotids, and Larynx) doses across the three groups (*p* > 0.02).

A variety of plan quality parameters were calculated for all target volumes. Dose metrics, such as, dose conformity, homogeneity and coverage were analysed. For each of the three groups, the dose coverage at D98% and D50% for the PTVs were comparable. The D2% dose levels for the PTV65 and PTV65-GTV increased. All dose levels associated with PTV54 remained largely unaffected by the dose escalation regimens. The conformity indices for PTV65 and PTVAll reveal comparable coverage for all three groups (Table 4).

Merlotti et al., [24] recommended that no more than 20% of PTV65 and no more than 1 cc of the tissue outside PTV65 should receive > 110% of the prescription dose. In keeping with this, we found that on average no more than 13.5% of PTV65-GTV received > 110% of 65 Gy and 0.2 cc of the Body–PTV65 structure received 71.5 Gy for the higher escalated group III. Furthermore, the volume of the body exposed to low dose levels (V_6__Gy_, V_12__Gy_, V_24__Gy_ and V_48__Gy_) are similar across the three groups.

Olteanu et al., [19] compiled their excellent body of work on dose escalation for H&N cancer and attempted to identify certain risk factors that influence late grade 4 mucosal ulcers. The most pertinent risk factor that can be applied directly to this study is a dosimetric threshold above which, one can assume, the occurrence of late grade 4 mucosal ulcers will most likely occur. Adopting the previously mentioned threshold of no more than 1.75 cc of GTV should receive > 84 Gy, we ensured agreement with this constraint. Every plan met this dosimetric threshold, which served as a valuable planning clinical goal.

While only pertinent OARs were focused on in this study given their proximity to the PTVs, the dose fall-off outside the GTV boost volume along with PTV65 doses were used as a surrogate of mucosal doses. By evaluating the ratios, Body/GTVx and doses to structures such as Body-GTV, Body-(GTV + 5 mm) and Body-(GTV + 10 mm), we found that the 107% volume of the conventional prescribed dose, 65 Gy, increased albeit to clinically acceptable levels inside the PTV65 volumes as described by the HDFI’s. These margins could be reasonably interpreted as safety margins ensuring that escalated dose levels to the PTV65-GTV and the OAR volumes were not excessively high. At a distance of 10 mm outside the GTV, the V107%_(65__Gy)_ had decreased by 87.6% (2.1 cc) for the 73 Gy group and 93.9% (3.4 cc) for the 82 Gy group plans. These values can be reassuringly compared to 3.5 cc at a 10 mm distance for the 65 Gy group. Although there may be an increased volume of 107% up to 10 mm outside the GTV, De Felice et al., reported that the majority of recurrences occur within the GTV-CTV 10 mm margin [25].

We found the escalated plans to be less modulated than the standard, control plans. This may be attributed to how the optimisation objective priorities were modified during planning. As we allowed the dose to escalate for the group II and group III plans, our cost functions weren’t penalised to the same extent resulting in fewer objective priorities driving the optimiser. Nevertheless, the recorded modulation values for all plans were within the range accepted in standard clinical plans.

IMRT planning techniques, such as VMAT when compared to conventional planning can offer greater protection of normal tissue adjacent to tumours while still delivering targeted therapeutic radiotherapy doses. VMAT when conflated with advanced treatment planning solutions, such as RapidPlan™ and MCO can result in highly conformal dose distributions in areas specifically defined by the radiation oncologist and targeted by the treatment planner. The simultaneous integrated boost (SIB) technique is a strategy that enables simultaneous planning and irradiation of multiple target volumes to varying dose levels [26, 27]. Our approach intended to intensify the dose of radiation to the GTV, while maintaining dosimetrically acceptable PTV coverage, OAR doses and overall clinically acceptable treatment plans.

While very promising, this dose escalation study has some limitations. One potential unintended effect of the use of MCO is the possible increase in dose to other OARs not considered in this study. The volume of the body receiving 48 Gy (V_48__Gy_) was, however, used as a surrogate indicator for scatter dose and did not increase significantly. All treatment plans were carried out on a single pre-treatment CT scan for each patient. Patients with OPSCC can undergo significant weight loss and changes in anatomy over their several weeks of treatment. Further work will examine the impact these changes may have on the feasibility of dose-escalation to GTV in the 2^nd^ half of treatment, in particular the doses to OARs and surrounding PTVs.

## Conclusions

This planning study in OPSCC showed the feasibility of significant dose-escalation to the GTV while maintaining clinically acceptable PTV65-GTV and OAR doses. Analysis of the plan quality metrics indicate that this can be achieved without a compromise to plan quality. This work lays the foundations for the clinical investigation of response adaptive dose-escalated radiotherapy in poor prognosis OPSCC.

## Data Availability

The datasets used and analysed during the current study are available from the corresponding author on reasonable request.

## Abbreviations

ALPO: Average leaf pair opening
CI: Conformity index
C(RT): Chemoradiotheapy
CTV: Clinical target volume
DTA: Distance to agreement
DVH: Dose volume histogram
EQD2: Equivalent dose in 2 Gy per fraction
GTV: Gross tumour volume
H&N: Head and neck
HDFI: High-dose falloff index
HI: Homogeneity index
HPV: Human papillomavirus
HNSCC: Head and neck squamous cell carcinoma
IMRT: Intensity modulated radiotherapy
LRC: Loco-regional control
MCO: Multi-criteria optimisation
MF: Modulation factor
OAR: Organs at risk
MLC: Multi-leaf collimator
MU: Monitor units
OPSCC: Oropharangeal squamous cell carcinoma
PTV: Planning target volume
PO: Photon optimiser
PRV: Planning risk volume
PTV65: Main planning target volume
PTV54: Low-risk planning target volume
PTVAll: PTV65 plus PTV54
QA: Quality assurance
RP: RapidPlan
SIB: Simultaneous integrated boost
TPS: Treatment planning system
VMAT: Volumetric arc therapy
